# 

**Published:** 1899-06

**Authors:** 


### PERSONALS.

Dr. E. H. Brown (McK. '97) is employed by the United States Army as veterinarian to the port of Manila. He reports the prevalence of rinderpest and foot-and-mouth disease among the cattle and buffaloes of Luzon.

Dr. J. B. Jones, graduate of the New York College of Veterinary Surgeons, class of '97, who has been practising at Milford, Del., has located at Atlantic City, N. J.

Dr. A. Crowforth, of Lockport, N. Y., is the owner of several fast trotting-horses, and will track them the coming season.

Dr. Tait Butler, of Mississippi, addressed the Louisiana State Agricultural Society in January on "The Fattening of Cattle for Market a Profitable Industry for the Louisiana Farmer."

Dr. Harri Dell, of Wabash, Indiana, conducts the Wabash Red Cross Veterinary Hospital in connection with his outside practice, both of which continually improve.

Dr. Harry Walters, of Wilkesbarre, Pa., who recently retired from practice in that city, has under completion at Kirkville, Missouri, laboratories for the conducting of physiological and psychological investigation.

Dr. John H. McNeal, of the Bureau of Animal Industry, formerly of Buffalo, has been transferred to the meat-inspection service at Brooklyn, N. Y.

F. W. Benteen, V.S., of Charleston, S. C., son of the late General Benteen, United States Army, was among the one hundred and one new second lieutenants recently appointed by President McKinley.

R. B. Corcoran, Sr., V.S., Eighth cavalry, United States Army, has been granted a six months' leave of absence.

Dr. Edward W. O'Connor, of Philadelphia, graduate of the Veterinary Department of the University of Pennsylvania, class of 1897, received the degree in human medicine at the Medico-Chirurgical College in May.

Dr. Harry Lukes, of Springfield, Mass., will make a trip to Europe for a period of six to eight weeks.

Dr. M. Jacob, of the class of '99, has accepted the position of Resident Veterinarian at the Veterinary Department of the University of Pennsylvania.

Dr. John M. Parker, of Haverhill, Mass., member of the Board of Live-Stock Commissioners, started for his native country, Scotland, in June, to be absent until September. Dr. Parker has been absent from his native heath for fourteen years.

Dr. E. M. Rauck, of Philadelphia, veterinarian to the H. K. Mulford Company biological laboratories, is conducting a series of experiments looking to the production of a snake-venom antitoxin. His methods for obtaining the snake venom from the numerous specimens of deadly snakes, with which the laboratory abounds, are unique and interesting.

Dr. C. S. McKenna has recently located at Derrick City, Pa.

Dr. E. E. Tower, of Montrose, Pa., was a visitor to Philadelphia in June relative to matters pertaining to the State Livestock Sanitary Board.

Dr. W. E. Wight, formerly of Delaware, Ohio, and who recently graduated at the McKillip Veterinary College, has located for practice in Allegheny, Pa.

Dr. Leonard Pearson was a visitor to Greater New York in the latter part of May.

Dr. S. J. J. Harger, of Philadelphia, was one of the veterinarians to the Baltimore horseshow in May.

Dr. A. Jasme, of Savannah, Ga., has been suffering with malaria for some time.

Dr. F. W. Culver, of Longmont, Colo., conducts large food and sale stables in connection with his veterinary practice.

Drs. H. D. Gill, of New York; W. H. Ridge, of Trevose, and C. J. Marshall, of Philadelphia, officiated as veterinarians at the Philadelphia horseshow May 30th, 31st, and June 1st, 2d, and 3d.

Dr. F. H. Mackie, of Fair Hill, Md., was a visitor to Philadelphia in April.

